# Bacteria on display—can we, and should we? Artistically exploring the ethics of public engagement with science in microbiology

**DOI:** 10.1093/femsle/fny101

**Published:** 2018-04-24

**Authors:** Nicola J Fawcett, Anna Dumitriu

**Affiliations:** 1 Nuffield Department of Medicine, University of Oxford, UK; 2 Oxford University Hospitals NHS Foundation Trust, UK; 3 University of Hertfordshire, UK; 4 Brighton and Sussex Medical School, UK

**Keywords:** public engagement, art, ethics, health and safety, communication, *trans*-disciplinary

## Abstract

The field of microbiology presents unique opportunities, and accompanying challenges, for artistic collaborations. On one hand, artistic works enable exploration of the aesthetics and of issues in biomedical science and new technologies, and draw in new, non-scientific audiences. On the other hand, creating art with microbes requires rigorous consideration of health and safety. Artists working in this field, known as Bio Art, tend to want to push the boundaries of what is possible or ‘known’, and work with new biomedical tools as they become available. However, when an artist's proposed work is raising novel questions where the risks are not fully understood, who should decide if the benefits outweigh the consequences? The reflections of an art-collaborating scientist are related. Also, considered is how close working relationships between disciplines can enable new ethical frameworks that consider these decisions, respecting artists’ endeavours as a beneficial form of research in its own right, and even learning from the rich perspectives of artists to broaden reflections on the practice of science.

## Teaser

An experience of science/art collaboration is discussed, together with the ethical issues that arise, and how they can be addressed in novel *trans*-disciplinary methods.

## INTRODUCTION

Anna Dumitriu, our ‘Artist in Residence’ is next to me in the lab, spraying silk with ‘DNA-Away’. We are discussing the safety of bacterially impregnated quilting, made using faecal samples from my own research project studying antimicrobial resistance. We ask ‘would you touch it?’, ‘would you lick it?’ and ‘can horizontal gene transfer occur from autoclaved, irradiated organisms?’. We’d done a literature search, which concluded it may be theoretically possible (Yap, Goldsmith and Moore 2013), hence the ‘DNA-Away’. Even then, I ask—should we do this?

Dumitriu began working with our research group in 2010 as part of our public engagement strategy, stemming from an earlier collaboration with one of the project leaders. She has completed residencies with a range of institutions, from Public Health England working with *Staphylococcus aureus* and DNA sequencing, to the University of California, Irvine working with synthetic biology. Her works have featured internationally. We have now collaborated over a number of years; these conversations form a key part of the creative process.

The training process in microbiology rightly emphasises a rigorous approach to health and safety—‘if in doubt, do the safest thing’, leading one to question why you would take potentially pathogenic bacteria out of the lab ‘for art’. In this piece, we summarise, based on personal experience and a small selection of the wider literature, questions we have found of use in considering the display microbiology–art collaborations. We consider the potential benefits that such collaborations can produce, some reasons why the display of art made from ‘real bacteria’ may make a piece more effective, and how we can address risk. Finally, we describe a *trans*-disciplinary project, which aims to explore how to weigh up these ethical issues and involve multiple stakeholders in discussion.

**Key Questions in displaying microbiological Bio Art**.

What are the overall aims and potential benefits of the piece?

Do these aims require, or are enhanced by, the use of the ‘real thing’?

If there are risks, how can they be minimised, and how do they compare to existing public displays?

How do the potential benefits of this artwork compare to the potential risks, and who should be involved in this evaluation?

### Aims and potential benefits

#### Stimulating personal reflection and interest

It is flattering to think of oneself as the scientist, guardian of knowledge and paternalistically responsible. Equally, it is easy to view artists as caring only about shock and impact, needing fences that scientists impose on them to create artwork which is ‘responsible’. I have found that the process of creation and collaboration has challenged these assumptions and has led to a more open view of inclusion of other viewpoints in decision-making, in both art and scientific research.

The projects have also been undeniably enjoyable, sparking a renewed appreciation for the bacteria I work with. Mehmet Berkmen, collaborator with artist Maria Penil noted a similar effect, saying, ‘There is a sense of aesthetics in our work and how we present our work. It is difficult to do and appreciate without art’ (Chan-Laddaran 2015).

#### Exploring new artistic areas

For the artist, there are additional aims—to ask unanswered questions, to pioneer new areas, to seek originality and how to create resonant and meaningful works of art. In Dumitriu's work, she also explores something she terms ‘the bacterial sublime’, an aesthetic sensation that draws together feelings of awe, terror and the inability to fully comprehend notions including complexity and scale (key properties of bacteria) (Dumitriu 2014). The sublime was memorably described by JF Lyotard as ‘the straining of the mind at the edges of itself and at the edges of its conceptuality’ (Lyotard 1991).

#### Education and engagement

Previous work has shown how artistic working in microbiology may enhance classroom learning, (Charkoudian *et al.*^2010^) and improve course outcomes (Adkins, Rock and Morris 2018). It can also serve as a link to non-scientific audiences, promoting microbiology research and engagement with subjects like antimicrobial resistance (Price 2015). Engagement via lay summaries, lectures and posters are effective at summarising information, but I have found they fail to address learning as an experience, driven by emotion, inspiration, beauty and curiosity. A key aim of collaborative art works, in my view, is to create a piece that inspires that ‘Ooooh!..?’ moment. Something immediately compelling, curious, visually intriguing, that draws you in, and makes you want to know more. Artists can give a masterclass in this skill. Certainly more people have asked me about bacterial culture techniques at art events than science ones. Rather than telling people what you think they should learn, we are inspiring curiosity, and they are asking you what they want to know.

#### Exploring ethics of emerging biotechnologies

To the wider community, Bio Art (where the artist works with live tissues, living organisms and life processes) has a track record in stimulating wider discussion about the ethics of new biotechnologies. Eduardo Kac's ‘GFP Bunny’ and Kathy High's ‘HLA-B27’ provoked wide debate on the ethics of transgenic animals (Stracey 2009). In a society where regulatory legislation is often reactive, Bio Art can act as a challenge, and, as Lawyer Lori B. Andrews notes in her Essay ‘Art as a Public Policy Medium’, play a wider role, stimulating society to confront wider social implications and develop policies for dealing with biotechnologies (Andrews 2007).

### What is the value in seeing the actual bacteria, the ‘real thing’?

**Figure 1.**
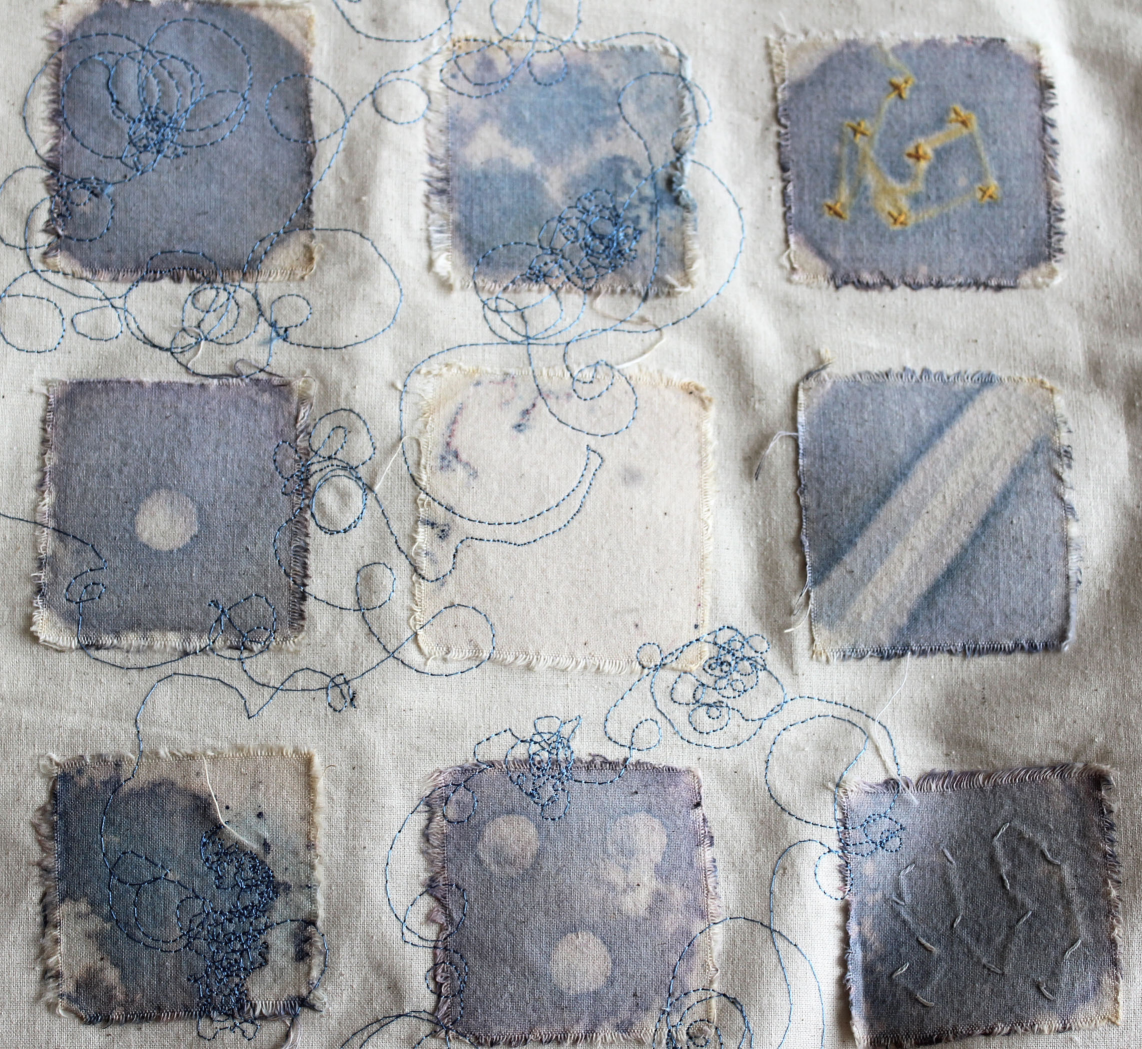
The MRSA Quilt. The quilting squares were added to Methicillin-resistant *Staphylococcus aureus* cultures on chromogenic agar. Standard antibiotic susceptibility testing equipment was used to create the patterns, together with other known antimicrobial pigments and dyes. The whole work was autoclaved prior to display. It serves as a tactile, aesthetic ‘conversation piece’, as microbiological techniques are described to explain how the effects are achieved. More information on this piece and more can be found at www.normalflora.co.uk.

**Figure 2.**
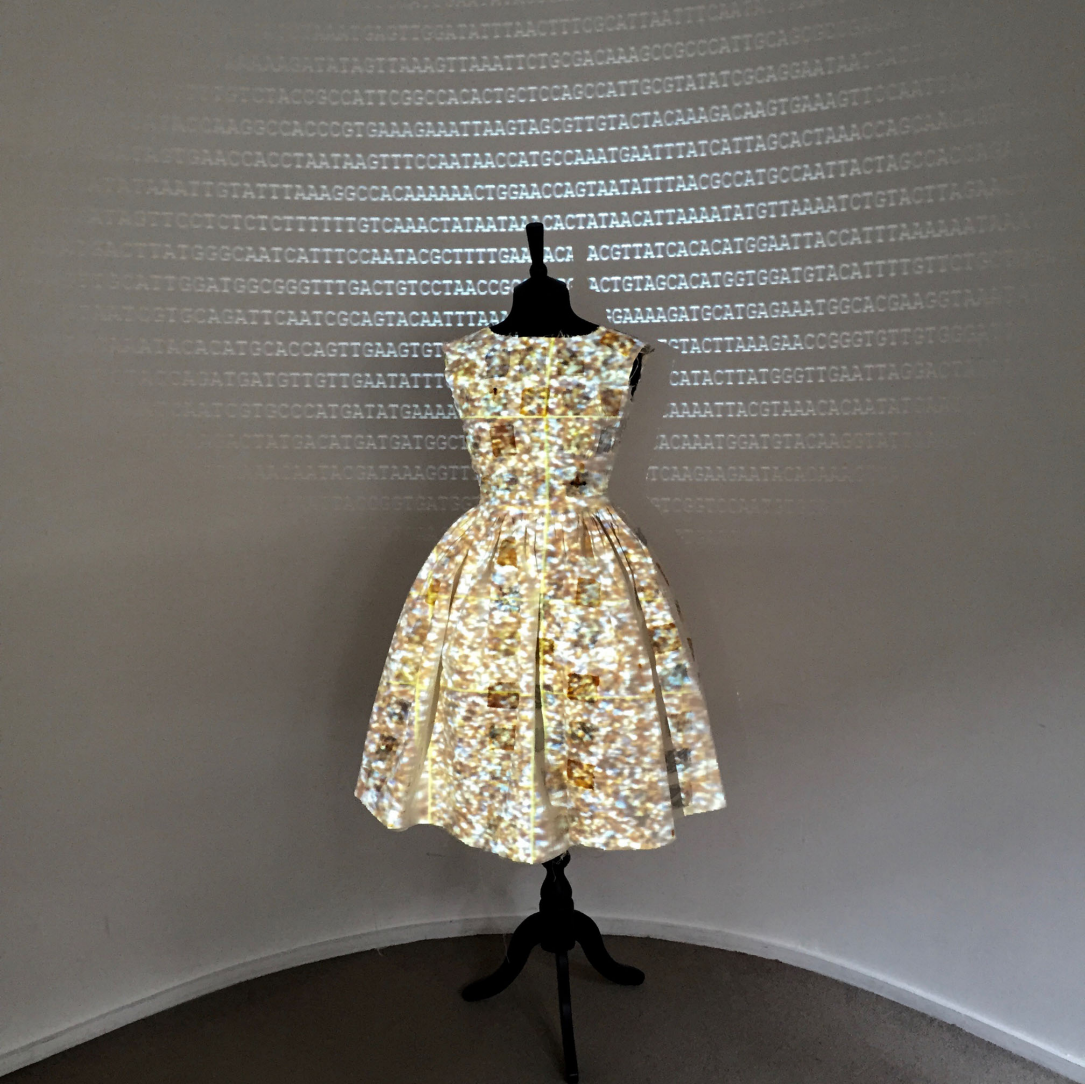
Sequence Dress. The dress was created using material impregnated and patterned using *Staphylococcus aureus* culture on chromogenic agar. DNA from *S. aureus* cultured from Dumitriu's body was sequenced using an Illumina Miseq, and the light output from the flow cells captured and mapped digitally onto a dress. The DNA sequence was projected behind. The piece has been exhibited internationally, and serves to highlight new technologies in the field of Microbiology. www.normalflora.co.uk.

One of Dumitriu's hallmarks is using viable bacteria to create her pieces. The ‘MRSA Quilt’, for instance, is made with fabric squares grown with the bacteria and autoclaved (Fig. 1). ‘Sequence Dress’ features the DNA of *S. aureus* cultured from Dumitriu's own body (Fig. 2). In Bio Art—Altered Realities, William Myers defines Bio Art as either utilizing biology as an artistic medium or seeking to alter the meaning of biology in its outcome (Myers 2015), as opposed to representing biological entities using non-biological media.

There are many successful art/science collaborations that do not use ‘live’ bacteria to create the final display piece. From microbiologically inspired watercolours (Banks 2016) to 3D-printed virus particles and photography of ‘bacterial drawings’ on agar (Kmietowicz 2015; Madhusoodanan 2016), much can be achieved by creative representations of microbiology. Working with Dumitriu and using often-unpredictable biological material has made me appreciate the skill required to integrate this uncertainty into the aesthetic and story of the piece. I have certainly found the sense of awe when you explain that a piece displayed was made ‘using real superbugs’ is palpable. The philosopher Immanuel Kant suggests that the experience of the sublime (Dumitriu's special interest) is something that takes place in the mind of the viewer (Kant 1799), rather than being situated in a ‘sublime object’ as was proposed by Burke (1756). Perhaps we become desensitised in the lab. The gallery space, the realm of art, has the ability to help specialist and non-specialist alike, to experience bacteria anew.

The importance of inspiring this level of awe cannot be overstated—every scientist can probably relate a multitude of experiences that instilled in them the love of their subject. I vividly remember seeing the Natural History Museum in London as a child, the ‘real’ dinosaur bones that roamed the earth millennia ago, and how their bones told stories about evolution. The value of the authentic to the overall experience is compelling.

### Minimising, considering, comparing and relativising risk

I have often found myself feeling that if there is residual theoretical risk, it is better not to go ahead. Yet it is worth standing back and putting into perspective—at museums around the country there is much publically accessible material with associated risk: bones, glass lab equipment, mercury thermometers—all deemed appropriate for display in appropriate circumstances.

Far from the image of the artist as the irresponsible risk-taker, Dumitriu works with expert microbiologists to integrate compliance with health and safety standards into her work. She went through formal ethical review to display a sterilised human faecal transplant at the Eden Project. We have specific laboratory risk assessments for Bio Art creation and she undergoes the necessary vaccinations and training. Whilst other artists may have differing levels of expertise and focus, it is through collaborative working that this experience can be developed.

### Weighing up the relative potential risks and benefits—who and how?

If a piece presents complex ethical issues, especially in pioneering fields with associated knowledge limitations—who deserves a voice in the decision making? How do you ensure these voices are heard? The role of patient/public representatives on scientific ethics panels is well recognised. If it is the wider public that stand to benefit, and equally, be put at risk, then their opinions must be heard alongside those of microbiologist, artist and ethics specialist. To this end, Dumitriu is the lead artist in the innovative project ‘Trust me, I’m an Artist: Developing Ethical Frameworks for Artists, Cultural Institutions and Audiences Engaged in the Challenges of Creating and Experiencing New Art Forms in Biotechnology and Biomedicine in Europe’ (Farsides, Dumitriu and Bureaud 2016). This project aims not just to assist decision-making, but also to open up opportunities for new collaborations, giving confidence that challenging ethical issues can be done responsibly and productively through joint work between artists, scientists, curators, galleries and museums. This *trans*-disciplinary approach (a ‘unity of intellectual frameworks beyond the disciplinary perspectives’ (Marilyn 1991)) has already fed into a number of Dumitriu's works and provides a thought-provoking blueprint in how these ethical issues might be addressed.

## CONCLUSION

The sense of awe, respect and curiosity that microbiology can inspire is often hard to adequately translate, yet can be explored by specialist and non-specialist alike using artistic works. These can serve as vivid, aesthetic experiences that stimulate the wish to know more, and that ask searching, sometimes uncomfortable questions, challenging scientists, artists and participants alike. We suggest that innovative, *trans*-disciplinary methodologies are required to push boundaries whilst maintaining robust ethical approaches.

In the era where we may be facing a ‘post-antibiotic apocalypse’, which scientists themselves struggle to adequately understand, let alone communicate, art/science collaborations can explore how we can share our experiences and stories. The consequences of research in contemporary microbiology, new discoveries in genomics, sequencing and synthetic biology can be explored artistically and responsibly to evoke and enable interaction with the sublime world of bacteria.
